# Population genetic structure and demographic of *Spodoptera eridania* (Lepidoptera: Noctuidae), an emerging soybean pest in Brazil

**DOI:** 10.1093/jisesa/ieaf092

**Published:** 2025-10-28

**Authors:** Renato J Horikoshi, Frederico Nanini, Davi de S Fernandes, Geraldo U Berger, Patrick M Dourado, Ramiro F L Ovejero, Alberto S Corrêa

**Affiliations:** Bayer Crop Science, São Paulo, SP, Brazil; Departamento de Entomologia e Acarologia, Escola Superior de Agricultura “Luiz de Queiroz”, Universidade de São Paulo, Piracicaba, SP, Brazil; Departamento de Entomologia e Acarologia, Escola Superior de Agricultura “Luiz de Queiroz”, Universidade de São Paulo, Piracicaba, SP, Brazil; Departamento de Entomologia e Acarologia, Escola Superior de Agricultura “Luiz de Queiroz”, Universidade de São Paulo, Piracicaba, SP, Brazil; Bayer Crop Science, São Paulo, SP, Brazil; Bayer Crop Science, São Paulo, SP, Brazil; Bayer Crop Science, São Paulo, SP, Brazil; Departamento de Entomologia e Acarologia, Escola Superior de Agricultura “Luiz de Queiroz”, Universidade de São Paulo, Piracicaba, SP, Brazil

**Keywords:** insect distribution, genetic diversity, Integrated Pest Management, secondary pest

## Abstract

*Spodoptera eridania* (Stoll) is a key pest of soybean crops in Brazil. However, information about the physiology, behavior, and ecology of *S. eridania*, including its genetic variability within the agricultural landscape, remains scarce. In this study, we conducted an exploratory analysis of the genetic diversity, population structure, and demographic patterns of *S. eridania* in Brazil. A *cytochrome c oxidase I* (COI) gene fragment of 866 bp was sequenced from 89 *S. eridania* individuals collected in Brazilian soybean macroregions. We identified 33 COI haplotypes with high haplotype diversity and moderate nucleotide diversity distributed throughout the country. The genetic relationships among COI haplotypes show a recent divergence with no evidence of distinct haplogroups. *Spodoptera eridania* collected on soybean crops showed a lack of population genetic structure associated with soybean macroregions or Brazilian states. Overall, our data suggest that *S. eridania* populations are undergoing demographic and spatial expansion in Brazil, with an increasing effective population size in the last 200 years. This study provides the first insights into the population diversity and demography of *S. eridania* in the Americas, shedding light on the dynamics and evolution of this species and further supporting integrated pest management strategies in Brazilian soybean crops.

## Introduction

Soybean is the main crop planted in Brazil and one of the most important sources of oil and protein worldwide (USDA 2022). In the 2023/2024 season, the area planted with soybean in Brazil reached 45.9 M ha, with an estimated production of 147 M tons (CONAB—Companhia Nacional de Abastecimento 2025). This scenario was quite different decades ago, when soybean was a minor crop in Brazil, with only about 1.3 M ha planted in 1970 (Cattelan and Dall’Agnol 2018). Over the past 50 years, several significant changes have occurred in Brazilian agriculture, including the adoption of no-tillage cultivation, the expansion of planting in the Cerrado region, the development of new crop varieties, the implementation of multi-crop systems with at least 2 cropping seasons per year, and the use of genetically modified plants (Fatoretto et al. 2017, Cattelan and Dall’Agnol 2018). These changes directly affect the insect population dynamics on soybean crops, where species considered primary decreased their abundance, and other species initially secondary emerged as key pests (Horikoshi et al. 2021, Saldanha et al. 2024).

*Spodoptera* species (Lepidoptera: Noctuidae), considered major and widespread Lepidopteran pests, have significantly threatened soybean crops in Brazil (Horikoshi et al. 2021, Kergoat et al. 2021). The most notorious among them is the fall armyworm, *Spodoptera frugiperda* (Smith) (Lepidoptera: Noctuidae), a polyphagous pest with massive importance in maize crops (Blanco et al. 2016, Goergen et al. 2016, Fatoretto et al. 2017, Jing et al. 2020). Population genetics studies of *S. frugiperda* reveal the presence of host-adapted strains associated with maize and rice plants in the Americas (Pashley and Martin 1987, Nagoshi and Meagher 2022). The strains exhibited a high genetic structure in Brazil’s landscape, as the maize strain is more prevalent in maize and cotton crops, while the rice strain is common in rice crop areas (Nagoshi et al. 2007, Silva-Brandão et al. 2018). However, when evaluating the intraspecific genetic variability of each strain, we see that the populations show a low genetic structure and high gene flow in Brazil, promoting that adaptive alleles, eg associated with resistance to insecticides and Bt transgenic plants, spread rapidly in the landscape (Arias et al. 2019, Boaventura et al. 2020, Ishizuka et al. 2023).

Another native *Spodoptera* species, *Spodoptera eridania* (Stoll) (Lepidoptera: Noctuidae), is a polyphagous pest distributed across the Americas, feeding on a wide range of hosts, including vegetables, fruits, and crops (Montezano et al. 2014, Specht and Roque-Specht 2016). This species may become increasingly economically significant, particularly in soybean–cotton farming in Brazil’s Cerrado region, due to its ability to use both crops as hosts (Santos et al. 2005, Horikoshi et al. 2021). Currently, the transgenic *Bt* soybean expressing Cry1Ac is effective in controlling many Noctuidae pests on soybean crops, with 82% adoption in Brazil in the season 2023/2024, but it is not effective in suppressing *Spodoptera* species, including *S. eridania*, increasing the relative abundance of these species on soybean crops in the last years (Horikoshi et al. 2021, Godoy et al. 2022, Kynetec 2024). Thus, neurotoxic insecticides are the main strategies for controlling *S. eridania*, despite studies on biological control agents for this pest control (Bortolotto et al. 2014, Scudeler et al. 2019, Machado et al. 2020).

Information about the physiology, behavior, and ecology of *S. eridania* is scarce, as well as genetic variability and its distribution within populations in the agricultural landscape, which have never been estimated. Population genetics studies can identify genetic strains and population connections, providing insights into the species’ evolution and ecological dynamics (Dong et al. 2021, Lai et al. 2024). Particularly for agricultural pests, these studies provide valuable information for developing targeted and sustainable control measures, which can help improve insect resistance programs to insecticides and Bt crops, as well as their sustainable management (Hereward et al. 2017, Nagoshi and Meagher 2022, Neiva et al. 2023).

Despite this critical role in Integrated Pest Management and Insect Resistance Management, the genetic diversity and population structure of important agricultural pests remain poorly understood. This knowledge gap includes *S. eridania*, a species with a broad geographic distribution and a diverse host range but lacking information on its genetic diversity, population structure, and demographic patterns. Thus, we conducted an exploratory analysis of the genetic diversity, population structure, and demographic patterns of *S. eridania* in Brazil based on *cytochrome c oxidase I* (COI) gene, providing the first data on *S. eridania* population diversity and demography in the American continent and offering insights into the dynamics of this species on soybean crops.

## Materials and Methods

### Insect Collection and DNA Extraction

**Fig. 1.**
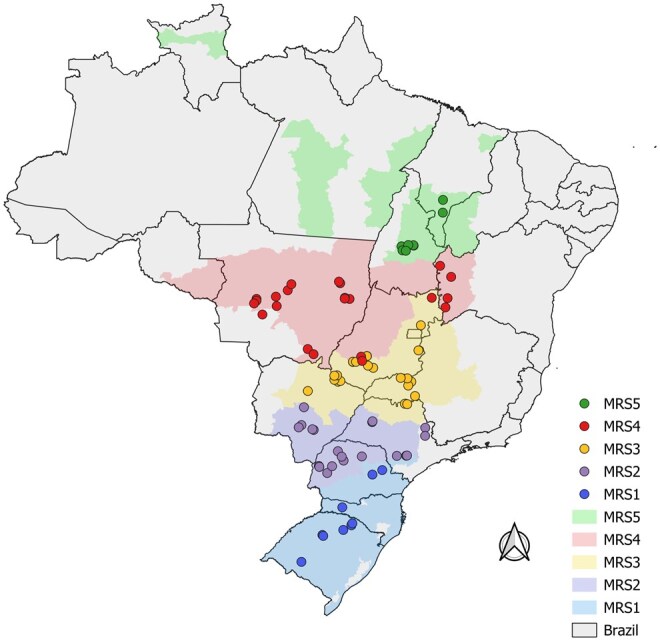
Sample site collections for *Spodoptera eridania* individuals on soybean in Brazil. Color areas and points indicate the soybean macroregions where individuals were sampled. The map was generated using QGIS 3.34.

Eighty-nine *S. eridania* were sampled in the 2020/2021 season from soybean fields located in 15 Brazilian states, representing all 5 soybean macroregions (MRS1, MRS2, MRS3, MRS4, and MRS5) in Brazil (Kaster and Farias 2012) (Fig. 1 and Supplementary Table S1). Larvae were collected directly on soybean plants and added to tubes with propylene glycol. After being in the laboratory, the species were identified, and larvae were transferred to 99.5% ethanol and stored in a freezer at −20 °C. DNA was extracted using the modified CTAB protocol (Doyle and Doyle 1987).

### PCR Amplification and Sequencing

Mitochondrial COI gene fragment was amplified by polymerase chain reaction (PCR) using the primers SpoF1 (Forward) (5′-TGTAGAAAATGGAGCAGGAAC-3′) and SpoR1 (Reverse) (5′-CTGAATATCGACGAGGTATACC-3′), which were designed from COI gene sequence of *S. frugiperda* found on Popset 310617289 available at NCBI.

The PCR reactions were performed in 25 µl total volume: 2 µl of DNA (50 ng/µl); 19.25 µl Mili-Q water; 0.25 µl 10× PCR Buffer Mg^2+^ free (Thermo Fisher Scientific); 1.25 µl MgCl_2_ (50 mM) (Thermo Fisher Scientific); 0.125 µl dNTP (10 mM) (Sinapse Inc); 1 µl of each primer (5 µM); and 0.125 µl Platinum Taq DNA Polymerase (5 U µl^−1^) (Thermo Fisher Scientific). The program for the thermocycler to PCR amplification was 94 °C for 3 min for primary denaturation, followed by 35 cycles of 94 °C for 30 s, 53 °C for 45 s, and 72 °C for 2 min, with a final extension at 72 °C for 10 min. To confirm the PCR reaction, the amplified fragments were separated on an agarose electrophoresis gel stained with SYBR Safe (Life Technologies) and visualized under UV light. Then, the PCR products (amplicons) were purified using 2.0 µl EXO-SAP (Cellco Biotec) for each 5 µl of the amplicon. The purification was made in the thermocycler at 37 °C for 30 min, followed by 80 °C for 15 min. Afterwards, they were sent for Sanger sequencing. All sequences were manually edited using the software BioEdit (Hall 1999). After editing and aligning, the mitochondrial COI sequence length used for the analysis of *S. eridania* was 866 bp.

### Population Diversity and Structure

For population diversity analysis, the *S. eridania* individuals were grouped according to the geographical location of sampling in soybean macroregions. The number of haplotypes (*H*), haplotype diversity (*Hd*), nucleotide diversity (*π*), average number of nucleotide differences (*k*), and number of variable sites (*S*) were estimated using DNAsp v.6 (Rozas et al. 2017).

The genealogical relationship among COI haplotypes was reconstructed using a network of Median-Joining haplotypes in PopArt v1.7 software (Leigh and Bryant 2015). The genetic distance among haplotypes was calculated using MEGA 11 (Tamura et al. 2021) with the Kimura *2*-parameter model (Kimura 1980) and 5000 bootstrap repetitions.

Analysis of molecular variance (AMOVA) was performed at 2 hierarchical levels: grouping individuals by soybean macroregions, as initially described, and also by Brazilian states and regions. Analysis was performed using Arlequin with a parametric bootstrap (5000 replicates) and a 5% significance level (Excoffier et al. 2005).

### Population Demography

The Tajima’s *D* and Fu’s *Fs* neutrality tests were performed in Arlequin v3.1 software (Excoffier et al. 2005). For both tests, significance was determined with 5,000 permutations in coalescent simulations. Tests were conducted for the individuals grouped in soybean macroregions. Fu’s *Fs* statistics were significant at 5% when the *P*-value was <0.02, as indicated in the Arlequin v3.1 software manual (Excoffier et al. 2005). Significant negative values of Tajima’s *D* or Fu’s *Fs* indicate an abundance of low-frequency haplotypes, supporting the hypothesis of population expansion or purifying selection. In contrast, significant positive values support the hypothesis of a population bottleneck.

A mismatch distribution analysis using a spatial expansion model was performed to test the population spatial expansion hypothesis estimated using molecular distance-based 5,000 bootstrap repetitions. The sum of square deviation (*SSD*), raggedness index (*r*), and associated *P*-value were calculated using Arlequin (Excoffier et al. 2005). If the SSD *P*-value >0.05, the population expansion hypothesis cannot be rejected. A raggedness index *P*-value >0.05 indicates a good data fit to the model.

A Bayesian Skyline Plot was used to reconstruct the demographic history of the species based on mitochondrial COI sequences in Beast v.1.8.4 software. We employed a strict molecular clock model to estimate the substitution rate, and coalescent tree priors were set to the constant size model. The insect molecular clock corresponds to 3.54% divergence per million years (Papadopoulou et al. 2010). Three independent runs of 100 million generations, sampling every 5000 steps, and 20% were discarded as burn-in. Convergence, effective sample size, and mean with 95% highest posterior density interval for divergence times were calculated in TRACER v.1.7.1.

## Results

### Population Diversity and Structure

An 866 bp region of the COI gene from *S. eridania* individuals was successfully sequenced and used for analysis (GenBank access numbers: PV440226 to PV440314). A total of 33 haplotypes were identified, 24 of which were unique to a single individual. The 4 most frequent haplotypes among the samples were H2, H5, H8, and H9, with a total frequency of 54% among the 89 individuals evaluated.

The overall haplotype diversity, nucleotide diversity, mean number of nucleotide differences, and number of variable sites were *Hd *= 0.922, *π* = 0.0035, *k *= 3.002, and *S *= 27, respectively. Haplotype diversity, nucleotide diversity, an average number of nucleotide differences, and the number of variable sites were similar among macroregions MRS2, MRS3, and MRS4 (*Hd* ranging from 0.913 to 0.938, *π* ranging from 0.00345 to 0.00384, *k* ranging from 2.990 to 3.326, and *S* ranging from 15 to 16). Lower values were observed for MRS1 (*Hd *= 0.873, *π* = 0.0024, *k *= 2.109, and *S *= 7) and MRS5 (*Hd *= 0.722, *π*  =  0.0019, *k *= 1.722, and *S *= 5), which may be influenced by the lower number of individuals collected in these macroregions (Table 1).

**Table 1.** Sample size (N), haplotypes (number), and genetic diversity indexes based on COI gene sequencing for *Spodoptera eridania* according to the soybean macroregions

Geographic region group	Sample size (*N*)	Haplotypes (*n*)	Haplotype diversity (Hd)	Nucleotide diversity (π)	Average number of nucleotide differences (k)	Number of variable sites (S)
**MRS1**	11	H2(4), H8, H9, H21, H25, H28(2), H29	0.87	0.0024	2.10	7
**MRS2**	21	H2(2), H4, H5(2), H8(5), H9, H10(2), H13, H17, H23, H24, H26, H27, H30, H32	0.94	0.0035	2.99	15
**MRS3**	24	H2(5), H4(4), H5, H6, H7, H8(3), H9(2), H10, H11, H12, H14, H15, H16, H31	0.93	0.0038	3.32	16
**MRS4**	24	H1(1), H2(6), H3, H4(2), H5, H8(2), H9(4), H10, H18, H19, H20, H21(2), H22	0.91	0.0036	3.07	15
**MRS5**	9	H5(5), H8, H9, H20, H33	0.72	0.0020	1.72	5
**Pooled**	89	–	0.92	0.0035	3.00	27

**Fig. 2.**
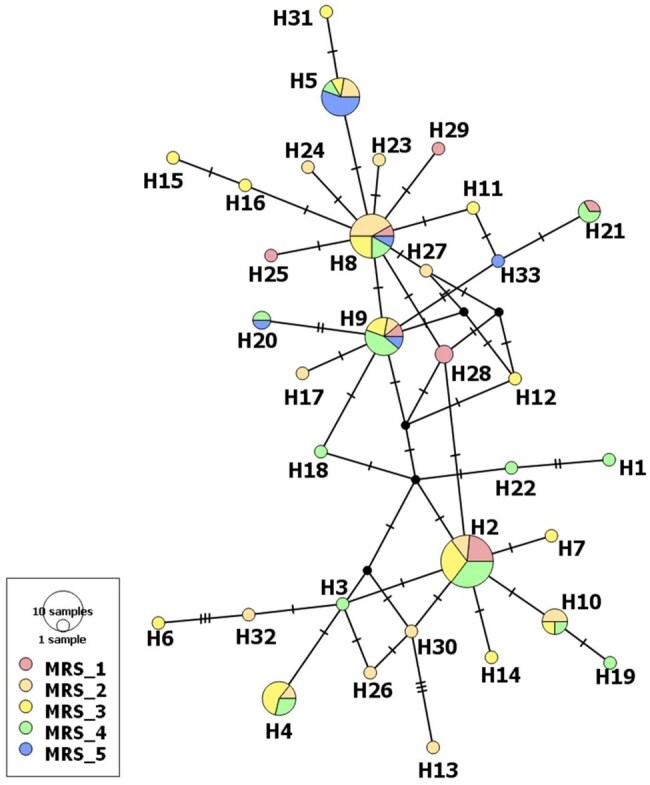
Haplotype network of *Spodoptera eridania* based on COI gene fragment. Haplotype circle size represents sample size, and dark circles represent missing haplotype. Tick marks reflect the number of mutation steps. Colors indicate the soybean macroregions where individuals were sampled.

The haplotype network analysis shows a low genetic distance among *S. eridania* haplotypes, showing recent genetic relationships among them. The most frequent haplotypes (H2, H5, H8, and H9) were widely distributed across all 5 macroregions (Fig. 2). The genetic distance (K2-P) among haplotypes ranged from 0.0015 to 0.0117.

The AMOVA grouping individuals into soybean macroregions revealed that most of the variation (96.99% of the total variation) was attributed to differences within populations, with a low *Φ_ST_* of 0.03 (*P *= 0.01) among the soybean macroregions (Table 2). The AMOVA with 2 hierarchical levels tested the hypothesis that the population structure, considering Brazilian states, was not significant (*Φ_ST_* = 0.028, *P *= 0.07) (Table 2).

**Table 2.** Analysis of molecular variance (AMOVA) based on mitochondrial COI sequences for *Spodoptera eridania* grouped among (a) soybean macroregions and (b) Brazilian states

Source of variation	d.f.	Sum of squares	Variance components	Percentage variance	Fixation indices (*P*-value)
**(a) Grouped soybean macroregions**					
**Among macroregions**	4	2.76	0.014	3.01	*Φ_ST_* = 0.030 (*P *= 0.011)
**Within macroregions**	84	37.80	0.450	96.99	
**Total**	88	40.56	0.464		
**(b) Grouped Brazilian states**					
**Among states**	14	7.32	0.013	2.79	*Φ_ST_* = 0.028 (*P *= 0.070)
**Within states**	74	33.23	0.449	97.21	
**Total**	88	40.56	0.462		

### Demographic Statistics

Negative values were observed in both Tajima’s *D* and Fu’s *Fs* neutrality tests, although significance was only observed in Fu’s *Fs* test (Table 3), indicating population expansion or purifying selection on MRS2, MRS3, and MRS4. MRS1 and MRS5 were not significant for Fu’s *Fs* test (*P *> 0.02) (Table 3). Considering all individuals grouped in Brazil, we obtained high negative and significant values in Fu’s *Fs* test (−24.94, *P *< 0.001) (Table 3).

**Table 3.** Neutrality test and mismatch distribution analysis based on mitochondrial COI sequences for *Spodoptera eridania* sampled in Brazil

Geographic region group	Sample size	Tajima’s D test	Fu’s *Fs* test	SSD	r
**MRS1**	11	−0.47 (0.34)	−2.70 (0.02)	0.0037 (0.84)	0.045 (0.83)
**MRS2**	21	−1.03 (0.15)	−8.04 (<0.001)	0.0025 (0.70)	0.024 (0.81)
**MRS3**	24	−0.80 (0.23)	−6.16 (<0.01)	0.0068 (0.52)	0.035 (0.58)
**MRS4**	24	−0.83 (0.21)	−5.30 (<0.01)	0.0039 (0.70)	0.033 (0.66)
**MRS5**	9	−0.27 (0.41)	−1.19 (0.12)	0.0025 (0.94)	0.029 (0.99)
**Pooled**	89	−1.33 (0.08)	−24.94 (<0.001)	0.0007 (0.87)	0.020 (0.78)

Neutrality and mismatch distribution tests are significant at *P-*value < 0.05.

The non-significant values of *SSD* (*P *> 0.52) and *r* (raggedness) (*P *> 0.58) support the hypothesis of spatial expansion of *S. eridania* populations in the 5 soybean macroregions analyzed and in Brazil (Table 3).

**Fig. 3.**
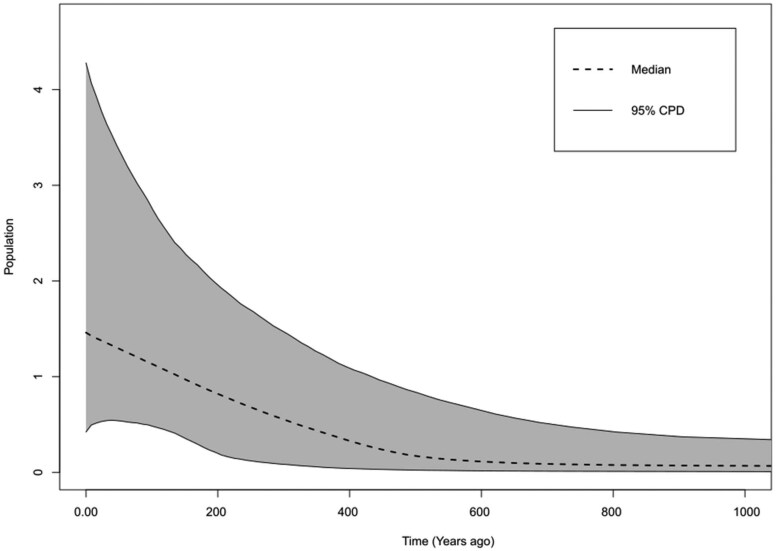
Bayesian skyline plot (BSP) for *Spodoptera eridania* based on COI gene fragment. The figures represent the effective population size as a function of time. Dashed lines represent the median BSP estimate, and gray areas are the 95% posterior density limits.

The Extended Bayesian Skyline Plot (*BSP*) analysis revealed demographic equilibrium, accompanied by an expansion of effective size in Brazil approximately 600 years ago, with a higher intensification of effective number increase over the last 200 years (Fig. 3).

## Discussion

High haplotype and moderate nucleotide diversity were observed for *S. eridania* sampled from soybean crops across all macroregions. The diversity index values found for *S. eridania* using the COI marker were similar to those reported for *S. frugiperda* in Brazil (Arias et al. 2019). When comparing these diversity indices with those described for other Noctuidae pests in soybean crops, *S. eridania* exhibits mitochondrial diversity comparable to or higher than that of different species (Domingues et al. 2012, Silva et al. 2020, Perini et al. 2021).

Despite its high mtDNA haplotype diversity, *S. eridania* exhibits a recent relationship among haplotypes, with no differentiation between haplogroups or strains. Additional *S. eridania* collections from distinct host plants other than soybean would be necessary to determine whether host-associated speciation is present, as seen in *S. frugiperda*, which has distinct corn and rice strains in the Americas (Pashley and Martin 1987, Dumas et al. 2015, Silva-Brandão et al. 2018). Furthermore, *S. eridania* is distributed throughout the entire American continent. Comparing Brazilian *S. eridania* populations with those in Central and North America, regions where this species also occurs, could help test for isolation by distance and/or isolation by resistance among continental populations. It could reveal an older genetic relationship among *S. eridania* populations, showing distinct haplogroups or strains between the American continents, as described for other insect pests with a wide distribution on the continent (Nagoshi et al. 2017, Parish et al. 2017, Francischini et al. 2019, Moraes et al. 2023).

Our results suggested a lack of population genetic structure, based on COI data, for *S. eridania* in Brazil, with *Φ_ST_* = 0.030, indicating that the prominent source of variation was found within the population. This becomes evident when we observe the most frequent mtDNA haplotypes, which are widely distributed across all macroregions. A lack of population genetic structure could lead to a panmixia hypothesis of *S. eridania* on soybean crops in Brazil’s territory. *Helicoverpa zea* (Boddie) (Lepidoptera: Noctuidae) is an example of a crop pest in panmixia within the South and North American continents, as suggested by SSR and SNP markers (Leite et al. 2017, North et al. 2024), probably due to the high dispersion capacity described in *Helicoverpa* species induced by climatic factors and host availability (Dourado et al. 2021, Paula-Moraes et al. 2024). However, despite wide insect collections nationwide, this is still an initial insight, as mitochondrial markers might fail to detect small-scale and recent genetic structures in the landscape (Wirth and Bernatchez 2001, Dong et al. 2021). Thus, additional studies using nuclear markers, based on SNP or SSR data, are needed to confirm the high gene flow and its associated factors, such as high moth dispersion capacity and/or recent genetic drift driven by demographic expansion or selection.

The Fu’s *Fs* and Bayesian Skyline Plot suggest that *S. eridania* populations have been undergoing demographic expansion in the Brazilian territory over the last 600 years, with the majority of this growth occurring in the last 200 years. The mismatch distribution analysis also indicated a spatial expansion of *S. eridania* in soybean areas. The demographic and spatial expansion of *S. eridania* may be attributed to landscape changes resulting from climatic and anthropogenic actions over the last few centuries. Thus, we hypothesize that the spatial and demographic expansion of *S. eridania’*s population was accelerated by the agricultural expansion and intensification in the last century, accompanied by the exploration of the Cerrado biome and the implementation of new cultivation technologies and strategies. Along this path, changes in insect demography were expected as new environments were occupied, primarily by species for which crops are a highly sustainable host, such as *S. eridania*. Similar outcomes were observed for other insect pests in Brazil, such as *Chrysodeixis includens* (Walker) (Lepidoptera: Noctuidae), *Crocidosema* sp. Zeller (Lepidoptera: Tortricidae), and stink bugs (Hemiptera: Pentatomidae) (Silva et al. 2020, Moraes et al. 2023, Singh et al. 2023, Fernandes et al. 2024). These changes, when associated with the nuclear genome, may eventually lead to varying levels of insect adaptation (McCulloch and Waters 2023). However, this requires further investigation into *S. eridania*.

Our results enhance the understanding of the population dynamics of *S. eridania* in Brazil, revealing high genetic diversity, a lack of population genetic structure, and signs of population expansion in soybean areas. This species is increasing its importance in soybean crops, resulting in the investment of new technologies (eg Bt soybean crops and entomopathogenic agents) and management strategies for its control (Godoy et al. 2022, Sampaio et al. 2024). Further studies employing genomic approaches with SNP markers are necessary to gain a deeper understanding of the gene flow and the evolutionary processes associated with the local adaptation mechanisms of *S. eridania* in the agricultural landscape and to inform control strategies. Knowledge of the geographic distribution of population diversity and the evolutionary processes of this pest across the country is essential for refining pest management recommendations, mainly when the objectives are to implement pest control strategies that are more sustainable and have a lower environmental impact.

## Supplementary Material

ieaf092_Supplementary_Data
