# Supplementary material for: Population genetic structure and demographic of Spodoptera eridania (Lepidoptera: Noctuidae), an emerging soybean pest in Brazil
Source: J Insect Sci. 2025 Oct 28;25(5):ieaf092. doi: 10.1093/jisesa/ieaf092 (PMC12568758; doi:10.1093/jisesa/ieaf092)
Supplement: ieaf092_Supplementary_Data [file ieaf092_supplementary_data.zip › Supplementary Table 1.docx]

**Population genetic structure and demographic of *Spodoptera eridania* (Lepidoptera: Noctuidae), an emerging soybean pest**

Supplementary Table 1. *Spodoptera eridania* (Lepidoptera: Noctuidae) insect collection on soybean areas in Brazil and their haplotype based on *cytochrome c oxidase I* (COI) gene fragment.

| **City** | **State** | **Soybean macroregion** | **Code** | **mtDNA haplotype** | **Latitude** | **Longitude** |
| --- | --- | --- | --- | --- | --- | --- |
| Passo Fundo | RS | 1 | 20RS02A1 | H28 | -28,29128841 | -52,33364786 |
| Sananduva | RS | 1 | 20RS04A1 | H28 | -27,93135038 | -51,79828011 |
| Sananduva | RS | 1 | 20RS05A1 | H02 | -27,99343928 | -51,81539755 |
| Sananduva | RS | 1 | 20RS06A1 | H02 | -27,87411824 | -51,75798504 |
| Cruz Alta | RS | 1 | 21RS23B1 | H09 | -28,61138400 | -53,66733600 |
| Cruz Alta | RS | 1 | 21RS24B1 | H29 | -28,66987600 | -53,61644300 |
| Cruz Alta | RS | 1 | 21RS24B2 | H21 | -28,67061800 | -53,61285000 |
| Rosário do Sul | RS | 1 | 21RS26B2 | H02 | -30,32632700 | -55,00477100 |
| Xanxerê | SC | 1 | 21SC01A1 | H02 | -26,84736400 | -52,39794700 |
| Piraí do Sul | PR | 1 | 21PR03A2 | H25 | -24,47219200 | -49,85135900 |
| Tibaji | PR | 1 | 21PR06A2 | H08 | -24,75864000 | -50,47768700 |
| Cafelândia | PR | 2 | 20PR08B1 | H26 | -24,65824280 | -53,35864140 |
| Terra Roxa | PR | 2 | 20PR13B1 | H02 | -24,21105149 | -53,89513277 |
| Palotina | PR | 2 | 21PR12B2 | H27 | -24,27068500 | -53,86008300 |
| Palotina | PR | 2 | 21PR12B1 | H02 | -24,26732500 | -53,86340100 |
| Peabiru | PR | 2 | 21PR18B2 | H23 | -23,86478800 | -52,31511000 |
| Goioerê | PR | 2 | 21PR20A2 | H24 | -24,22461900 | -53,05005000 |
| Paraíso do Norte | PR | 2 | 20PR21B2 | H08 | -23,26586300 | -52,67952300 |
| Jussara | PR | 2 | 21PR21B1 | H10 | -23,61361900 | -52,43433564 |
| Londrina | PR | 2 | 20PR31B2 | H10 | -23,60086065 | -51,15001655 |
| Itaí | SP | 2 | 21SP01A1 | H32 | -23,56499700 | -48,91737000 |
| Casa Branca | SP | 2 | 20SP04A1 | H30 | -21,77088372 | -47,10466898 |
| Mogi Guaçu | SP | 2 | 21SP04B1 | H04 | -22,28665550 | -47,11541997 |
| Angatuba | SP | 2 | 21SP08A1 | H08 | -23,55310420 | -48,31773230 |
| Angatuba | SP | 2 | 21SP08A2 | H05 | -23,54538930 | -48,27237410 |
| Birigui | SP | 2 | 21SP15A2 | H05 | -21,40880200 | -50,49471300 |
| Birigui | SP | 2 | 21SP16B1 | H08 | -21,35781100 | -50,47138400 |
| Rio Brilhante | MS | 2 | 20MS07B2 | H09 | -21,60112200 | -55,00265800 |
| Rio Brilhante | MS | 2 | 20MS01B2 | H13 | -21,90830800 | -54,22257800 |
| Campo Grande | MS | 2 | 21MS05B1 | H08 | -20,46219300 | -54,84470000 |
| Rio Brilhante | MS | 2 | 21MS11B1 | H08 | -21,86619200 | -54,30676900 |
| Maracaju | MS | 2 | 21MS15A1 | H17 | -21,74472700 | -55,18037300 |
| São Gabriel do Oeste | MS | 3 | 20MS12B2 | H14 | -19,42448500 | -54,59617400 |
| Chapadão do Sul | MS | 3 | 20MS14B1 | H16 | -18,83180300 | -52,67229600 |
| Chapadão do Sul | MS | 3 | 20MS13B2 | H15 | -18,78416200 | -52,51943300 |
| Chapadão do Sul | MS | 3 | 20MS16B1 | H04 | -18,71096900 | -52,90383400 |
| Guaíra | SP | 3 | 21SP11A1 | H04 | -20,23591300 | -48,35583800 |
| Guaíra | SP | 3 | 21SP11B1 | H02 | -20,23591300 | -48,35523800 |
| Guaíra | SP | 3 | 20SP12A2 | H09 | -20,23838700 | -48,40777500 |
| Guaíra | SP | 3 | 20SP06A2 | H31 | -20,26388069 | -48,25491680 |
| Uberaba | MG | 3 | 21MG01B1 | H11 | -19,74747377 | -47,76005923 |
| Araguari | MG | 3 | 20MG02B1 | H12 | -18,83209600 | -47,99156300 |
| Uberlândia | MG | 3 | 20MG06B1 | H09 | -19,07684000 | -48,19228500 |
| Araguari | MG | 3 | 20MG07A2 | H08 | -18,61856900 | -48,24327400 |
| Tupaciguara | MG | 3 | 21MG09A1 | H02 | -18,58865433 | -48,67376327 |
| Montividiu | GO | 3 | 20GO13B1 | H10 | -17,19326300 | -50,82623400 |
| Chapadão do Céu | GO | 3 | 20GO01B1 | H05 | -18,42793200 | -52,72902100 |
| Formosa | GO | 3 | 21GO18B1 | H02 | -15,23099120 | -47,38756165 |
| Chapadão do Céu | GO | 3 | 20GO02B2 | H04 | -18,44684400 | -52,90600000 |
| Cristalina | GO | 3 | 21GO04B1 | H04 | -16,83263272 | -47,54034147 |
| Jataí | GO | 3 | 20GO05B1 | H07 | -17,56878900 | -51,71945600 |
| Jataí | GO | 3 | 20GO05B2 | H06 | -17,55620700 | -51,71982500 |
| Jataí | GO | 3 | 20GO06B1 | H08 | -17,56196000 | -51,52339500 |
| Santa Helena  de Goias | GO | 3 | 20GO08B1 | H02 | -17,94752500 | -50,42491000 |
| Rio Verde | GO | 3 | 20GO10B2 | H02 | -17,80648100 | -50,76199300 |
| Montividiu | GO | 3 | 20GO13B2 | H08 | -17,20123000 | -50,83214300 |
| Montividiu | GO | 4 | 20GO12B1 | H02 | -17,23466200 | -51,19536000 |
| Montividiu | GO | 4 | 20GO11B2 | H09 | -17,50422000 | -51,13209700 |
| Jaborandi | BA | 4 | 21BA13B1 | H03 | -14,08928735 | -45,81742566 |
| Barreiras | BA | 4 | 19BA311 | H04 | -11,43703098 | -46,15823518 |
| Barreiras | BA | 4 | 21BA01B1 | H01 | -12,14228238 | -45,45319919 |
| Correntina | BA | 4 | 21BA10B1 | H02 | -13,50195891 | -45,68113008 |
| Correntina | BA | 4 | 21BA10B2 | H02 | -13,47748593 | -46,68931545 |
| Querência | MT | 4 | 20MT1131 | H02 | -12,54647450 | -52,53109210 |
| Querência | MT | 4 | 20MT1151 | H09 | -12,44572650 | -52,61332590 |
| Sorriso | MT | 4 | 20MT801 | H08 | -13,00042790 | -55,87823740 |
| Sorriso | MT | 4 | 20MT821 | H18 | -12,61058650 | -55,64540230 |
| Campo Novo  do Parecis | MT | 4 | 20MT911 | H08 | -13,66460920 | -57,87561610 |
| Campo Novo  do Parecis | MT | 4 | 20MT92A1 | H19 | -13,54847600 | -57,87146640 |
| Sapezal | MT | 4 | 21MT10A1 | H21 | -13,40343878 | -56,63544874 |
| Canarana | MT | 4 | 20MT1122 | H20 | -13,55185860 | -51,93501840 |
| Rondonópolis | MT | 4 | 21MT19B1 | H02 | -16,74716700 | -54,60414500 |
| Itiquira | MT | 4 | 21MT20B1 | H10 | -17,07760800 | -54,23017700 |
| Diamantino | MT | 4 | 21MT22B1 | H05 | -14,00412300 | -56,58404300 |
| Canarana | MT | 4 | 21MT31B1 | H22 | -13,47894900 | -52,23597600 |
| Canarana | MT | 4 | 21MT32B1 | H09 | -13,52824000 | -52,24696700 |
| Tangará da Serra | MT | 4 | 21MT41A1 | H21 | -14,54329000 | -57,49523700 |
| Campo Novo  do Parecis | MT | 4 | 20MT58A1 | H02 | -13,66483070 | -57,87559130 |
| Campo Novo  do Parecis | MT | 4 | 20MT912 | H09 | -13,66470950 | -57,87704440 |
| Nova Mutum | MT | 4 | 20MT971 | H04 | -13,83695710 | -58,02805360 |
| Balsas | MA | 5 | 21MA01B1 | H09 | -7,23301046 | -45,97516243 |
| Tasso Fragoso | MA | 5 | 21MA03B1 | H08 | -8,04406771 | -45,98628802 |
| Porto Nacional | TO | 5 | 20TO01A1 | H20 | -10,19305085 | -48,61977073 |
| Porto Nacional | TO | 5 | 20TO02B2 | H05 | -10,45005932 | -48,55815394 |
| Palmas | TO | 5 | 20TO03A1 | H05 | -10,14765203 | -47,84500494 |
| Palmas | TO | 5 | 20TO03B1 | H05 | -10,13515604 | -47,84992117 |
| Palmas | TO | 5 | 20TO03A2 | H05 | -10,12210626 | -47,86239797 |
| Palmas | TO | 5 | 20TO04B2 | H05 | -10,11163961 | -48,18874394 |
| Porto Nacional | TO | 5 | 21TO04B2 | H33 | -10,47522331 | -48,34421776 |
